# Emerging Roles of Plant DNA-Binding With One Finger Transcription Factors in Various Hormone and Stress Signaling Pathways

**DOI:** 10.3389/fpls.2022.844201

**Published:** 2022-05-13

**Authors:** Zemin Wang, Darren Chern Jan Wong, Zhengliang Chen, Wei Bai, Huaijun Si, Xin Jin

**Affiliations:** ^1^State Key Laboratory of Aridland Crop Science, Gansu Agricultural University, Lanzhou, China; ^2^College of Life Science and Technology, Gansu Agricultural University, Lanzhou, China; ^3^Division of Ecology and Evolution, Research School of Biology, The Australian National University, Acton, ACT, Australia

**Keywords:** transcription factor, Dof, hormonal signaling, transcriptional regulation, abiotic stresses

## Abstract

Coordinated transcriptional regulation of stress-responsive genes orchestrated by a complex network of transcription factors (TFs) and the reprogramming of metabolism ensure a plant’s continued growth and survival under adverse environmental conditions (e.g., abiotic stress). DNA-binding with one finger (Dof) proteins, a group of plant-specific TF, were identified as one of several key components of the transcriptional regulatory network involved in abiotic stress responses. In many plant species, Dofs are often activated in response to a wide range of adverse environmental conditions. Dofs play central roles in stress tolerance by regulating the expression of stress-responsive genes via the DOFCORE element or by interacting with other regulatory proteins. Moreover, Dofs act as a key regulatory hub of several phytohormone pathways, integrating abscisic acid, jasmonate, SA and redox signaling in response to many abiotic stresses. Taken together, we highlight a unique role of Dofs in hormone and stress signaling that integrates plant response to adverse environmental conditions with different aspects of plant growth and development.

## Introduction

Transcription factors (TFs) are proteins that bind to specific DNA motifs in the promoter regions of target genes, thereby regulating their transcription. In plants, on average 5% of the protein-coding genes encode TFs (Jin J. et al., 2014). Plant TFs were classified into 58 families (and some into six superfamilies) based on their DNA-binding domains (Jin J. et al., 2014; 2016). Many TFs such as Dof, WRKY, ERF, NAC, GRAS, and MYB (Dubos et al., 2010; Nakashima et al., 2012; Muller and Munne-Bosch, 2015; Hakoshima, 2018; Ruta et al., 2020) play a crucial role in plant signaling and regulatory networks relevant to abiotic/biotic stress responses and many developmental/physiological processes (Lindemose et al., 2013; Gupta et al., 2015; Ramirez et al., 2021; Wang Z. et al., 2021).

In particular, one plant-specific TF contains a highly conserved DNA-binding domain and has been designated the DNA-binding with one finger (Dof) domain (Yanagisawa, 1995; Yanagisawa and Schmidt, 1999). The first Dof protein, ZmDof1 was identified to contain the CX_2_CX_21_CX_2_C protein motif in maize (Yanagisawa and Izui, 1993). The evolutionary analysis of Dofs among plant species has been studied extensively in model plants such as Arabidopsis and *Oryza sativa* (Lijavetzky et al., 2003; Khan et al., 2021) but also non-model systems like apple (Zhang Z. et al., 2018), physic nut and castor bean (Zou and Zhang, 2019), grape (Wang Z. et al., 2021) and between multiple plant species (Noguero et al., 2013; Ma et al., 2015). Generally, the Dof gene family contains a highly conserved Dof domain at the N-terminus of approximately 50 residues in length, an oligomerization site, a transcription regulation domain and a nuclear localization signal. Most Dofs have only one type of DNA binding region and oligomerization region, although some lack a transcription regulation domain or a specific DNA binding region (Krohn et al., 2002). Dof DNA-binding activities have been analyzed by different *in vitro* and *in vivo* approaches revealing that all the Dof TFs tested bind to a DOFCORE element (5′-(A/T)AAAG-3′) (Yanagisawa, 2016). In Arabidopsis, a systematic study of 529 TFs using a DNA affinity purification-sequencing (DAP-seq) technique revealed that all 39 Dof TFs evaluated had the aforementioned promoter binding preferences (O’Malley et al., 2016). Similar observations were also reported using independent *in vitro* assays in Arabidopsis (Imaizumi et al., 2005; Corrales et al., 2017) but also other plants such as potato (Kloosterman et al., 2013) and tomato (Corrales et al., 2014; Ewas et al., 2017).

It is well-known that the Dof domain also mediates protein-protein interactions (Krohn et al., 2002). In maize, Dof1 not only self-associates but interacts with another Dof protein, Dof2 (Yanagisawa and Izui, 1993; Cavalar et al., 2003; Yanagisawa, 2004). Interaction with other TF families such as Dof–bZIP (Vicente-Carbajosa et al., 1997) and Dof–MYB interaction (Diaz et al., 2002) were also observed. The C-terminal motifs of the Dof domain are also pivotal for various protein-protein interactions. For example, some cycling Dof factors (CDF) proteins typically contain specific domains in their C-terminal region such as the clock gene GIGANTEA (GI) and FLAVIN BINDING KELCH REPEAT F-BOX PROTEIN 1 (FKF1) -binding domains (Renau-Morata et al., 2020) that are known to participate in the control post-translational regulation by protein-protein interactions (Imaizumi et al., 2005; Kloosterman et al., 2013; Corrales et al., 2014). Nonetheless, it is noteworthy that factors such as the location of binding and the interaction with other factors such as chromosomal high-mobility group proteins (HMGBs) (Krohn et al., 2002; Cavalar et al., 2003) might determine the capacity of DNA binding and thus, transcriptional control at precise sites in the genome. Dofs are well known for their roles in growth and development processes such as seed germination (da Silva et al., 2016), flowering (Corrales et al., 2017; Liu X. et al., 2020), and leaf senescence (Shim et al., 2019; Zhuo et al., 2020). However, recent studies have also revealed pivotal roles of Dof TFs in various abiotic and biotic stresses but also hormone responses (Qin et al., 2019; Li et al., 2021; Ramirez et al., 2021; Wang P. et al., 2021). In this perspective article, we highlight 68 Dof proteins (as in Table 1 and Supplementary Figure 1) with potential roles in stress and hormone responses in a wide range of plants.

**TABLE 1:** Expression of *Dof* genes under abiotic stress and hormone.

A. Expression of Dof genes under abiotic stress							

		Upregulation under stress					
Species	*Dofs*	Salt	Drought	Cold	Heat	Light	References
*Arabidopsis*	*AtDof5.8*^a^**	Yes	Yes	—	—	—	He et al. (2015)
*Arabidopsis*	*AtDof1.7*	—	—	Yes	—	—	Carvallo et al. (2011)
*Arabidopsis*	*AtCDF1/2/3/4/5*	—	Yes	Yes	—	Yes	Corrales et al. (2017) and Renau-Morata et al. (2017)
*Brassica napus*	*BnCDF1^a^*	No	—	Yes	No	Yes	Xu and Dai (2016)
*Brassica rapa L.* ssp. *pekinensis*	*BraDof072*	No	Yes	Yes	Yes	—	Ma et al. (2015)
*Brassica rapa L.* ssp. *pekinensis*	*BraDof074*	Yes	Yes	Yes	Yes	—	Ma et al. (2015)
*Capsicum annuum*	*CaDof1/16*	Yes	—	—	Yes	—	Wu et al. (2016)
*Citrullus lanatus*	*ClDof5/29/35*	Yes	—	—	—	—	Zhou et al. (2020)
*Chrysanthemum morifolium*	*CmDof4*	Yes	Yes	No	Yes	—	Wen et al. (2016)
*Chrysanthemum morifolium*	*CmDof7*	Yes	Yes	Yes	Yes	—	Wen et al. (2016)
*Cleistogenes songorica*	*CsDof5/10*	Yes	—	Yes	Yes		Wang P. et al. (2021)
*Cleistogenes songorica*	*CsDof23*	Yes	—	Yes	No	—	Wang P. et al. (2021)
*Daucus carota* subsp. *sativus*	*DcDof-A-1*	No	No	No	Yes	—	Huang et al. (2016)
*Daucus carota* subsp. *sativus*	*DcDof-B-2*	Yes	Yes	Yes	Yes	—	Huang et al. (2016)
*Gossypium hirsutum*	*GhDof1*	Yes	—	Yes	—	—	Su et al. (2017)
*Jatropha curcas*	*JcDof1^a^*	—	—	—	—	Yes	Yang et al. (2010)
*Juglans regia*	*JrDof3^a^*	—	—	—	Yes	—	Yang et al. (2018)
*Malus domestica*	*MdDof2/3*	Yes	Yes	No	—	—	Zhang Z. et al. (2018)
*Malus domestica*	*MdDof1*	Yes	Yes	Yes	—	—	Zhang Z. et al. (2018)
*Medicago sativa*	*MsDof16*	No	No	Yes	—	—	Cao et al. (2020)
*Medicago sativa*	*MsDof14*	Yes	Yes	No	—	—	Cao et al. (2020)
*Oryza sativa*	*OsDof15^a^*	Yes	—	—	—	—	Qin et al. (2019)
*Phyllostachys edulis*	*PheDof6*	—	Yes	No	—	—	Cheng et al. (2018)
*Phyllostachys edulis*	*PheDof13*	—	No	Yes	—	—	Cheng et al. (2018)
*Phyllostachys edulis*	*PheDof12-1*	Yes	Yes	Yes	—	—	Liu et al. (2019)
*Pyrus bretschneideri*	*PbDof9.2*	—	—	—	—	Yes	Liu X. et al. (2020)
*Rosa chinensis*	*RchDof7/23*	Yes	Yes	—	—	—	Nan et al. (2021)
*Solanum commersonii*	*ScDof1.7*	—	—	Yes	—	—	Carvallo et al. (2011)
*Solanum lycopersicum*	*SlCDF1^a^*	Yes	Yes	Yes	No	Yes	Corrales et al. (2014)
*Solanum lycopersicum*	*SlCDF2^a^*	Yes	Yes	No	Yes	No	Corrales et al. (2014)
*Solanum lycopersicum*	*SlCDF3^a^*	Yes	Yes	Yes	No	Yes	Corrales et al. (2014) and Renau-Morata et al. (2017)
*Solanum lycopersicum*	*SlCDF4^a^*	Yes	Yes	Yes	Yes	No	Corrales et al. (2014)
*Solanum lycopersicum*	*SlCDF5^a^*	No	No	Yes	Yes	No	Corrales et al. (2014)
*Saccharum spontaneum*	*SsDof5/28*	Yes	Yes	Yes	Yes	—	Cai et al. (2020)
*Solanum tuberosum*	*StCDF1^a^*	—	Yes	—	—	—	Ramirez et al. (2021)
*Solanum tuberosum*	*StDof1.7*	—	—	Yes	—	—	Carvallo et al. (2011)
*Spinacia oleracea*	*SoDof3/15/22*	—	Yes	Yes	Yes	—	Yu H. et al. (2021)
*Triticum aestivum*	*TaDof1*	Yes	Yes	—	No	Yes	Shaw et al. (2009)
*Triticum aestivum*	*TaDof14*	No	Yes	—	No	No	Shaw et al. (2009)
*Triticum aestivum*	*TaDof26/96*	—	Yes	Yes	No	—	Liu Y. et al. (2020)
*Triticum aestivum*	*TaDof35*	—	No	No	Yes	—	Liu Y. et al. (2020)
*Tamarix hispida*	*ThDof14*	Yes	Yes	—	—	—	Yang et al. (2017)
*Vitis vinifera*	*VaDof17a/b/d*^a^**	—	—	Yes	—	—	Wang Z. et al. (2021)
*Vitis yeshanensis ‘Yanshan’*	*VyDof8*	Yes	Yes	Yes	—	—	Li et al. (2021)
*Zea mays* subsp. *mays*	*ZmDof06*	Yes	Yes	—	—	—	Chen and Cao (2014)
*Zea mays* subsp. *mays*	*ZmDof22/36*	Yes	No	—	—	—	Chen and Cao (2014)

B. Hormonal effects on the transcription of *Dof* genes								

	Induced expression							
Gene	Ethylene	ABA	Jasmonate	Salicylic acid	GAs	BRs	Auxin	References
*AtDof5.8^a^*	—	Yes	—	—	—	—	—	He et al. (2015)
*AtOBP1*	—	—	—	Yes	—	—	Yes	Chen et al. (1996) and Kang and Singh (2000)
*AtOBP2*	—	—	Yes	Yes	—	—	Yes	Kang and Singh (2000) and Skirycz et al. (2006)
*AtOBP3*	—	—	—	Yes	—	—	Yes	Kang and Singh (2000) and Kang et al. (2003)
*AtOBP4*		Yes	—	—	—	—	—	Rymen et al. (2017)
*ClDof3/5/20/29*	—	Yes	—	—	—	—	—	Zhou et al. (2020)
*CmDof3*	—	No	Yes	No	—	—	—	Song et al. (2016)
*CmDof12*	—	Yes	No	Yes	—	—	—	Song et al. (2016)
*CmDof16*	—	No	Yes	Yes	—	—	—	Song et al. (2016)
*CsDof5/24/36*	—	Yes	—	—	—	—	—	Wang P. et al. (2021)
*FaDof2*	—	No	—	—	—	—	Yes	Molina-Hidalgo et al. (2017)
*MaDof23/24/25*	Yes	—	—	—	—	—	—	Feng et al. (2016)
*MdDof3/5/9/15/18*	—	Yes	—	—	—	—	—	Zhang Z. et al. (2018)
*MsDof01/02/10/39*	—	Yes	—	—	—	—	—	Cao et al. (2020)
*OsDof3^a^*	—	—	—	—	Yes	—	—	Washio (2003)
*OsDof12*	—	—	—	—	—	Yes	—	Wu et al. (2015)
*OsDof24^a^*	No	No	Yes	—	—	—	—	Shim et al. (2019)
*PheDof12-1*^a^**	—	No	—	—	Yes	—	—	Liu et al. (2019)
*RcDof3/6*	—	Yes	—	—	Yes	—	—	Jin Z. et al. (2014)
*RcDof4/5*	—	Yes	—	—	No	—	—	Jin Z. et al. (2014)
*SsDof13/24*	Yes	Yes	—	—	Yes	—	Yes	Cai et al. (2020)
*SsDof29*	—	—	—	—	Yes	—	—	Cai et al. (2020)
*VvDof3*	—	—	Yes	Yes		—	—	Yu et al. (2019)

***(A)**: —, Not studied; OBP, Osmotin Promoter Binding Protein; COG, Cogwheel; DAG, Dof Affecting Germination; CDF, Cycling Dof Factors.*

***(B)**: —, Not studied; BRs, Brassinosteroids; GAs, gibberellins; OBP, Osmotin Promoter Binding Protein; CDF, Cycling Dof Factors.*

*^a^Dofs described as capable of binding to (T/A)AAAG.*

## Dof Associated With Abiotic Stress

Adverse environmental conditions such as extreme temperatures (e.g., freezing and heat), water status (e.g., drought and flood) and salinity affect the survival, growth and reproduction of plants. Expression of *Dofs* have been reported to be induced by salt, drought, cold, heat stress, and changes in light availability in various species (Table 1). One common theme is that *Dof* genes are often multi-abiotic stress-responsive: salt, drought, cold, and heat stress treatment (4 studies); drought, cold, and heat stress treatment (6 studies); salt and drought (7 studies), among others. In various plant systems (Ravindran et al., 2020; Li et al., 2021; Ramirez et al., 2021; Zhu et al., 2021), overexpression, silencing, and mutational experiments of *Dof* genes have also established their role in various abiotic stress tolerance (Supplementary Table 1).

### Dof Regulate Stress-Responsive Transcription Factors

**FIGURE 1:**
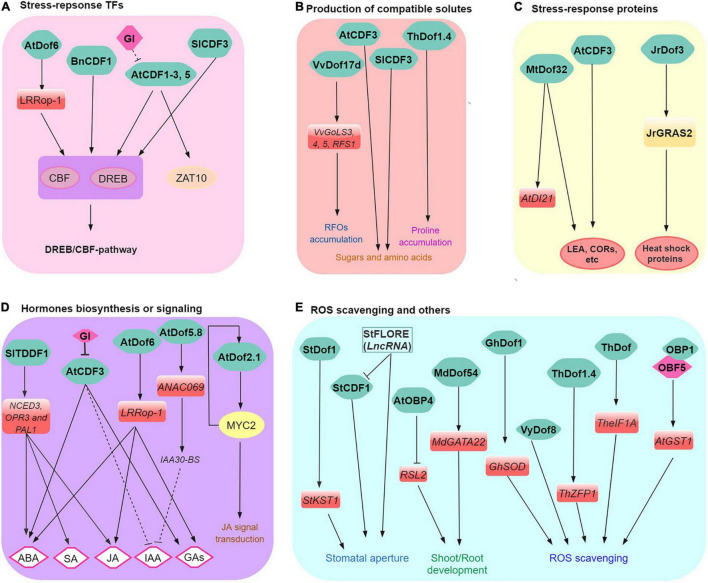
A simplified model of Dof functions in plant responses to abiotic stress. Dofs regulate **(A)** stress-related TFs (CBF, DREB, and ZAT10, etc., abiotic stress modules), **(B)** production of compatible solutes, **(C)** stress-related proteins (e.g., HSP and COR, etc.), **(D)** stress-related hormone biosynthesis or signaling, and **(E)** ROS scavenging and other biological processes. Positive and negative regulation are indicated by arrows and broken lines, respectively.

GIGANTEA is central in diverse signaling pathways, including circadian clock regulation, sugar, and light signaling pathways, photoperiodic and stress responses (including drought, salinity, and low temperature) (Han et al., 2013; Kim et al., 2013; Fornara et al., 2015; Park et al., 2016; Cao et al., 2020). Emerging results indicate a GI-CDF module where CDFs regulate genes involved in abiotic stress responses through regulation of the C-REPEAT BINDING FACTOR (CBF) regulon (Corrales et al., 2017; Xu et al., 2017; Shi et al., 2018; Tominaga et al., 2021). In some cases, CDFs have been identified as part of the CBF/DREB transcriptional regulatory network involved in controlling abiotic stress responses (Corrales et al., 2017; Renau-Morata et al., 2017; Liu et al., 2019; Ramirez et al., 2021). For example, Arabidopsis AtCDF3 regulates several stress-response TFs (e.g., CBFs, DREB2A, and ZAT10) which involve both GI dependent and independent pathways (Corrales et al., 2017; Renau-Morata et al., 2017). Similarly, overexpression of *Brassica napus BnCDF1*, which is homologous to Arabidopsis Cycling DOF Factor 1, promotes freezing tolerance via the regulation of different abiotic-stress-responsive genes such as *CBF1*, *CBF2*, *COR15A*, and *RESPONSIVE TO DESICCATION 29A* (*RD29A*; Xu and Dai, 2016). Thus the data suggest that regulation of *CBF* genes by Dof/CDFs might be the initial step in the abiotic stress response signaling cascade (Figure 1A).

### Dof Regulate the Production of Compatible Solutes

Galactinol synthases (GolS) and raffinose synthases (RFS) are closely related glycosyltransferases/hydrolases involved in the biosynthesis of raffinose family oligosaccharides (RFOs) that function as osmoprotectants that promote plant abiotic stress tolerance (Sprenger et al., 2018; Chai et al., 2019). In the Amur grape, transcriptomic analysis revealed that *GolS* and *RFS* are among the most cold-induced genes (Xin et al., 2013; Sun et al., 2018; Wang Z. et al., 2021). Overexpression and loss-of-function mutation (introduced by CRISPR/Cas9-mediated mutagenesis) of VaDof17d significantly induced and repressed expression of cold-induced GolS and RFS/RAF genes compared to wild-type plants, respectively. During cold stress, VaDof17d-ED loss-of-function mutants concordantly show poorer cold tolerance and decreased RFOs levels compared to normal plants (Wang Z. et al., 2021). These results highlight one alternative mechanism cold tolerance could be achieved in grapes (Figure 1B).

Furthermore, tomato *SlCDF1–5* genes were differentially induced in response to salt, drought, and heat stress (Corrales et al., 2014). Tomato plants overexpressing the *SlCDF3* showed enhanced biomass production and yield under salinity stress conditions (Renau-Morata et al., 2017). This has been attributed to higher sucrose, GABA, and asparagine in vegetative tissues of SlCDF3-overexpressing plants. These metabolites also act as cellular osmoprotectants in various abiotic stress conditions in plants (Mittler et al., 2011; Mittler, 2017). These reports point to a key role of Dof in the regulation of various compatible solutes under specific stress (e.g., cold and highly saline) conditions (Figure 1B).

### Dof Regulate Stress-Related Proteins

Heat shock proteins (HSPs) expression underlies adaptive responses to many environmental stress in plants. Briefly, HSPs function as molecular chaperones that encompasses many functions such as ensuring proper folding of other proteins but also assist in the deterrence, reduction, and/or degradation of stress-damaged proteins (Chen et al., 2018). Like HSPs, LATE EMBRYOGENESIS ABUNDANT (LEA) proteins also participate in acclimation and/or in the adaptive response to various stress (Battaglia et al., 2008). LEAs are a type of highly hydrophilic glycine-rich proteins with antioxidant, metal ion binding, membrane and protein stabilization, hydration buffering, and DNA and RNA interaction properties. In walnut, the heat-stress responsive JrGRAS2 TF regulates a multitude of heat shock proteins and is pivotal for heat stress response. Interestingly, walnut JrDof3 activates JrGRAS2, thereby reinforcing the regulation of HSP and heat stress response (Yang et al., 2018). In addition, *Medicago* MtDof32 plays a positive role in regulating the expression of stress-related genes (i.e., upregulation of DROUGHT-INDUCED 21, *AtDI21*; *SENESCENCE-ASSOCIATED GENE 21*; *AtCOR15A*) leading to enhanced osmotic and salt tolerances in Arabidopsis (Guo et al., 2021). These examples point to the role of Dof in the regulation of stress-related genes and potentially enhanced abiotic stress tolerance especially heat (Figure 1C).

### Dof Involved in Hormone Biosynthesis or Signaling

Coordinated regulation of multiple hormonal signaling pathways enables plants to respond and adapt to adverse environmental conditions by regulating the expression of TF genes in a finely tuned manner (Yang et al., 2019). Indeed, exogenous application of various phytohormones induces the expression of many Dof genes (Table 1) involved in hormone biosynthesis or signaling in many plants. Abscisic acid (ABA) is one key hormone that regulates plant responses to abiotic stresses (Tuteja, 2007). The grape *Vitis yeshanensis* VyDof8 showed multi-abiotic stress-responsive expression patterns. Overexpression of VyDof8 in tobacco (*Nicotiana benthamiana*) significantly enhanced ABA accumulation and drought tolerance during prolonged drought compared to control plants (Li et al., 2021).

In plants, jasmonate acid (JA) and its derivatives (e.g., MeJA and JA-Ile) also play diverse roles in response to abiotic stress (Chini et al., 2016; Wasternack and Feussner, 2018; Yang et al., 2019). With regards to the involvement of Dof proteins, Dof2.1 and MYC2 (MYC2, which encodes a central regulator of JA responses) form a feed-forward transcriptional loop (MYC2–Dof2.1–MYC2) that enhances jasmonate-induced leaf senescence in *Arabidopsis* (Zhuo et al., 2020). The integration of auxin and salt signals by ANAC069 in the regulation of Arabidopsis seed germination have been demonstrated (Park et al., 2011). Interestingly, subsequent studies revealed that Arabidopsis AtDof5.8 is the upstream regulator of ANAC069 that indirectly also mediates the regulation of salinity and osmotic stress tolerance (He et al., 2015, 2017). Salicylic acid has long been known to play a role in the induction of defense mechanisms in plants; however, its participation in abiotic stress signaling is slowly emerging (Siboza et al., 2014; Zhang L. et al., 2018; Yang et al., 2019). OBP1, OBP2, and OBP3 were initially identified as partners of OBF4 that interacts with salicylic acid-responsive ocs elements (Kang and Singh, 2000; Kang et al., 2003), thus potentially linking these Dofs in SA regulation.

In addition, Dof has been also reported to be involved in multiple hormonal pathways during abiotic stress (Figure 1D). For example, Dof6 induced *LRRop-1* negatively regulates abiotic stress responses during Arabidopsis seed germination by altering endogenous hormone levels of ABA, GA, and JA (Ravindran et al., 2020). In tomato, TDDF1 positively regulates gene expression patterns for *NCED3*, *OPR3*, and PAL1, the key genes of ABA, JA, and SA biosynthesis pathway under drought and salt stress (Ewas et al., 2017). Together, these examples reveal that Dofs mediates multiple hormone responses linked to abiotic and biotic stress responses in a wide range of plants.

### Dof Regulate Reactive Oxygen Species Scavenging and Others

Antioxidant genes are also involved in Dof-mediated stress responses (e.g., salt, drought) in some plants (Figure 1E). The reactive oxygen species (ROS, such as H_2_O_2_ and O^2–^) are considered to be both beneficial (e.g., signaling) and potentially damaging molecules which result in cell membrane damage by stimulating membrane lipid peroxidation, and even cell death (Mittler et al., 2011; Devireddy et al., 2020). Overexpression of *Tamarix hispida* ThDof was found to enhance drought tolerance in transgenic tobacco plants by indirectly regulating the expression of stress-related genes (including *ThSOD*; peroxidase *ThPOD* and glutathione peroxidase *ThGPX*), resulting in improved reactive ROS scavenging and tolerance to salt and osmotic stress (Yang et al., 2017). Additionally, transient overexpression and silencing of another Dof, *ThDof1.4* in *T. hispida* plants promoted and repressed the expression of *ThZFP1*, respectively. Together, ThDof1.4 and ThZFP1 form a transcriptional regulatory cascade and coordinately regulate the expression of *T. hispida* PODs, SODs, and proline biosynthesis-related genes leading to increased proline, enhanced ROS scavenging capacities. This finding point to a role of ThDof1.4 in salt and osmotic stress tolerance in *T. hispida* (Zang et al., 2017).

Moreover, CDFs are key elements that integrate plant responses to adverse environmental conditions with different aspects of plant growth control and development like photoperiodism or root/shoot growth (Figure 1E). In Arabidopsis, OBP4/Dof5.4 also plays a central role in integrating developmental and environmental signals by mediating the transcriptional repression of *RSL2* that ultimately contributes to the inhibition of root hair growth (Rymen et al., 2017). In cotton, overexpression of GhDof1 positively regulates *GhP5CS*, *GhSOD* and *GhMYB*, leading to improved salt tolerance and root growth over the wild-type plants (Su et al., 2017). Consistently, MdDof54 facilitated root development, stomatal conductance, transpiration, photosynthesis, and hydraulic conductivity, which lead to improved growth of apple trees under long-term drought stress (Chen et al., 2020).

## Dof Associated With Biotic Stress

In addition to the participation of Dofs in diverse abiotic stress responses and as a key regulatory hub of several phytohormone pathways (Figure 1 and Table 1), their roles in biotic stress response are slowly emerging. Phytocystatins (PhyCys) are a group of cystatin proteins in plants that specifically inhibits cysteine proteinases and peptidase that are often produced by pathogens to aid their colonization and proliferation in the host cells (Martinez et al., 2005). The role of Dof transcription factors in the regulation of PhyCys has been established in several plants for biotic stress tolerance. For example, rice *OsDof1* (Park et al., 2014) show stress-responsive induction in response to plant wounding and pathogen challenge. In barley (*Hordeum vulgare*), a Dof transcription factor, SAD binds to the pyrimidine box *in vitro* and activates transcription of a protease promoter (of a cystatin gene) in bombarded aleurone layers (Isabel-LaMoneda et al., 2003). Additionally, another Dof TF, BPBF also interacts with oligonucleotides containing Dof binding sites derived from the cystatin gene promoter *in vitro* (Mena et al., 2002; Diaz et al., 2005). Furthermore, tomato Dof, TDDF1 may increase biotic resistance by regulating Pathogenesis Related Protein 1 (PR1) expression, a well-known resistance marker gene induced in response to pathogen and herbivores attack (Ewas et al., 2017). In watermelon, 22 and 34 (of 36) *CsDof* genes were upregulated or downregulated in response to downy mildew and watermelon mosaic virus inoculation, respectively (Wen et al., 2016). In pepper, RNA-seq analysis showed temporal and pathogen-specific variation of *CaDofs* during development and response to multiple biotic stresses such as two TMV strains, PepMoV and *Phytophthora capsici* infection (Kang et al., 2016). These results indicated that Dof may be involved biotic stress response in a wide range of plants.

## Conclusion and Future Prospects

In this perspective article, we present supporting evidence that the Dof family appears to show more multifaceted roles than previously expected. We present evidence that Dofs act as a key regulatory hub of several phytohormone pathways, integrating abscisic acid, jasmonate, SA and redox signaling in response to many abiotic stresses. However, many gaps in our knowledge on Dofs and hormonal interaction (such as Dof with BR, GA, etc.), and the answers to the remaining questions are likely important to increase our understanding of stress responses and adaptation. Beyond hormonal-related abiotic stress regulation, some of Dofs also seem to regulate ROS-responsive gene expression, but more evidence of synergistic Dofs- and ROS-responsive genes are needed in multiple plant systems.

Since TFs play an important role in increasing stress tolerance and developmental responses in plants, they can be targeted for the generation of improved varieties using transgenic technology (Wang et al., 2016). Yet, large numbers of Dofs remain uncharacterized in plants. Nonetheless, as exemplified with the success in small studies targeting individual Dofs (Ramirez et al., 2021; Wang Z. et al., 2021) but also large-scale attempts to generate knockout lines for all Dof members within a single plant species such as all 30 *OsDofs* in rice (Huang et al., 2019; Yu L. et al., 2021), CRISPR/Cas system is poised to be an effective and flexible way to study the function of Dofs through the generation of functional (e.g., loss- and/or gain-of-function) mutants. Long non-coding RNAs (lncRNAs) are a heterogeneous class of regulatory transcripts that represent a diverse class of regulatory loci with roles in development and stress responses in plants. To date, only one report has reported a link between Dofs and lncRNA (Figure 1E), therefore, it is of importance to determine whether functional roles between Dofs and lncRNA are more widespread than what is currently known. Therefore, additional studies are required to fully understand the molecular mechanisms by which Dofs orchestrate metabolic homeostasis, stress responses, crop improvement and plant growth and development.

## Author Contributions

ZW, XJ, and HS conceptualized the theme of the article and compiled the manuscript. ZW and DW prepared the first draft after collecting literature. ZC and WB added the work related to hormone and biotic stress. All authors read and approved the manuscript.

## Conflict of Interest

The authors declare that the research was conducted in the absence of any commercial or financial relationships that could be construed as a potential conflict of interest.

## Publisher’s Note

All claims expressed in this article are solely those of the authors and do not necessarily represent those of their affiliated organizations, or those of the publisher, the editors and the reviewers. Any product that may be evaluated in this article, or claim that may be made by its manufacturer, is not guaranteed or endorsed by the publisher.
