# Supplementary material for: Emerging Roles of Plant DNA-Binding With One Finger Transcription Factors in Various Hormone and Stress Signaling Pathways
Source: Front Plant Sci. 2022 May 13;13:844201. doi: 10.3389/fpls.2022.844201 (PMC9165642; doi:10.3389/fpls.2022.844201)
Supplement: Supplementary file 1 [file Table_1.pdf]

Table S1. Abiotic/biotic stress response studied in transgenic plants overexpressing *Dofs*

CDF, Cycling Dof Factors; TDDF1, Tomato Dof Daily Fluctuations 1, OBP, OBF Binding Protein.

Gene	Transgenic Plants	Effect	Reference
AtCDF3[a]	Arabidopsis	Drought and cold tolerance	Corrales et al. (2017)
AtOBP2	Arabidopsis	Defense	Skirycz et al. (2006)
GhDof1[a]	*Gossypium hirsutum*	Salt and cold tolerance	Su et al. (2017)
MdDof54[a]	*Malus domestica*	Drought tolerance	Chen et al. (2020)
MtDof32	*Medicago truncatula*	Osmotic and salt tolerance	Guo et al. (2021)
OsDof1	*Oryza sativa*;	Wound-responsive	Park et al. (2014)
OsDof15[a]	*Oryza sativa*	Salt tolerance	Qin et al. (2019)
OsDof24[a]	*Oryza sativa*	Delays leaf senescence	Shim et al. (2019)
SlCDF3[a]	*Solanum lycopersicum*	Salt tolerance	Renau-Morata et al. (2017)
TDDF1[a]	*Solanum lycopersicum*	Salt and drought tolerance; Resistance to phytophthora infestans	Ewas et al. (2017)
StCDF1[a]	*Solanum tuberosum*	Drought tolerance	Ramirez et al. (2021)
ThDof1.4[a]	*Tamarix hispida*	Salt and drought tolerance	Zang et al. (2017)
VaDof17d[a]	*Vitis vinifera*	Cold tolerance	Zhu et al. (2021)

[a]Dofs described as capable of binding to (T/A)AAAG.

Refference

Chen, P., Yan, M., Li, L., He, J., Zhou, S., Li, Z., Niu, C., Bao, C., Zhi, F., Ma, F., Guan, Q. (2020). The apple DNA-binding one zinc-finger protein MdDof54 promotes drought resistance. Hortic Res 7 (1):195. doi:10.1038/s41438-020-00419-5

Corrales, AR., Carrillo, L., Lasierra, P., Nebauer, SG., Dominguez-Figueroa, J., Renau-Morata, B., Pollmann, S., Granell, A., Molina, RV., Vicente-Carbajosa, J., Medina, J. (2017). Multifaceted role of cycling DOF factor 3 (CDF3) in the regulation of flowering time and abiotic stress responses in Arabidopsis. Plant Cell Environ 40 (5):748-764. doi:10.1111/pce.12894

Ewas, M., Khames, E., Ziaf, K., Shahzad, R., Nishawy, E., Ali, F., Subthain, H., Amar, MH., Ayaad, M., Ghaly, O., Luo, J. (2017). The Tomato DOF Daily Fluctuations 1, TDDF1 acts as flowering accelerator and protector against various stresses. Sci Rep 7 (1):10299. doi:10.1038/s41598-017-10399-7

Guo, T., Wang, S., Zhang, T., Xu, L., Li, Y., Chao, Y., Han, L. (2021). Expression of the Medicago truncatula MtDof32 transcription factor regulates plant growth and enhances abiotic stress tolerances in transgenic Arabidopsis. Environmental and Experimental Botany 183. doi:10.1016/j.envexpbot.2020.104339

Park, SH., Lim, H., Hyun, SJ., Yun, D-W., Yoon, U-H., Ji, H., Kim, T-H., Eun, M-Y., Kim, Y-H., Lee, G-S. (2014). Wound-inducible expression of the OsDof1 gene promoter in a Ds insertion mutant and transgenic plants. Plant Biotechnology Reports 8 (4):305-313. doi:10.1007/s11816-014-0316-5

Qin, H., Wang, J., Chen, X., Wang, F., Peng, P., Zhou, Y., Miao, Y., Zhang, Y., Gao, Y., Qi, Y., Zhou, J., Huang, R. (2019). Rice OsDOF15 contributes to ethylene-inhibited primary root elongation under salt stress. The New phytologist 223 (2):798-813. doi:10.1111/nph.15824

Ramirez, GL., Shi, L., Bergonzi, SB., Oortwijn, M., Franco-Zorrilla, JM., Solano-Tavira, R., Visser, RGF., Abelenda, JA., Bachem, CWB. (2021). Potato CYCLING DOF FACTOR 1 and its lncRNA counterpart StFLORE link tuber development and drought response. Plant J 105 (4):855-869. doi:10.1111/tpj.15093

Renau-Morata, B., Molina, RV., Carrillo, L., Cebolla-Cornejo, J., Sanchez-Perales, M., Pollmann, S., Dominguez-Figueroa, J., Corrales, AR., Flexas, J., Vicente-Carbajosa, J., Medina, J., Nebauer, SG. (2017). Ectopic Expression of CDF3 Genes in

Tomato Enhances Biomass Production and Yield under Salinity Stress Conditions. Front Plant Sci 8:660. doi:10.3389/fpls.2017.00660

Shim, Y., Kang, K., An, G., Paek, N-C. (2019). Rice DNA-Binding One Zinc Finger 24 (OsDOF24) Delays Leaf Senescence in a Jasmonate-Mediated Pathway. Plant and Cell Physiology 60 (9):2065-2076. doi:10.1093/pcp/pcz105

Skirycz, A., Reichelt, M., Burow, M., Birkemeyer, C., Rolcik, J., Kopka, J., Zanor, MI., Gershenzon, J., Strnad, M., Szopa, J., Mueller-Roeber, B., Witt, I. (2006). DOF transcription factor AtDof1.1 (OBP2) is part of a regulatory network controlling glucosinolate biosynthesis in Arabidopsis. Plant J 47 (1):10-24. doi:10.1111/j.1365-313X.2006.02767.x

Su, Y., Liang, W., Liu, Z., Wang, Y., Zhao, Y., Ijaz, B., Hua, J. (2017). Overexpression of GhDof1 improved salt and cold tolerance and seed oil content in Gossypium hirsutum. Journal of plant physiology 218:222-234. doi:10.1016/j.jplph.2017.07.017

Zang, D., Wang, L., Zhang, Y., Zhao, H., Wang, Y. (2017). ThDof1.4 and ThZFP1 constitute a transcriptional regulatory cascade involved in salt or osmotic stress in Tamarix hispida. Plant Mol Biol 94 (4-5):495-507. doi:10.1007/s11103-017-0620-x

Zhu, X., Hong, X., Liu, X., Li, S., Yang, J., Wang, F., Yue, Y., Zhang, N., Si, H. (2021). Calcium-dependent protein kinase 32 gene maintains photosynthesis and tolerance of potato in response to salt stress. Scientia Horticulturae 285. doi:10.1016/j.scienta.2021.110179
