# Supplementary material for: Emerging Roles of Plant DNA-Binding With One Finger Transcription Factors in Various Hormone and Stress Signaling Pathways
Source: Front Plant Sci. 2022 May 13;13:844201. doi: 10.3389/fpls.2022.844201 (PMC9165642; doi:10.3389/fpls.2022.844201)
Supplement: Supplementary file 2 [file Image_1.pdf]

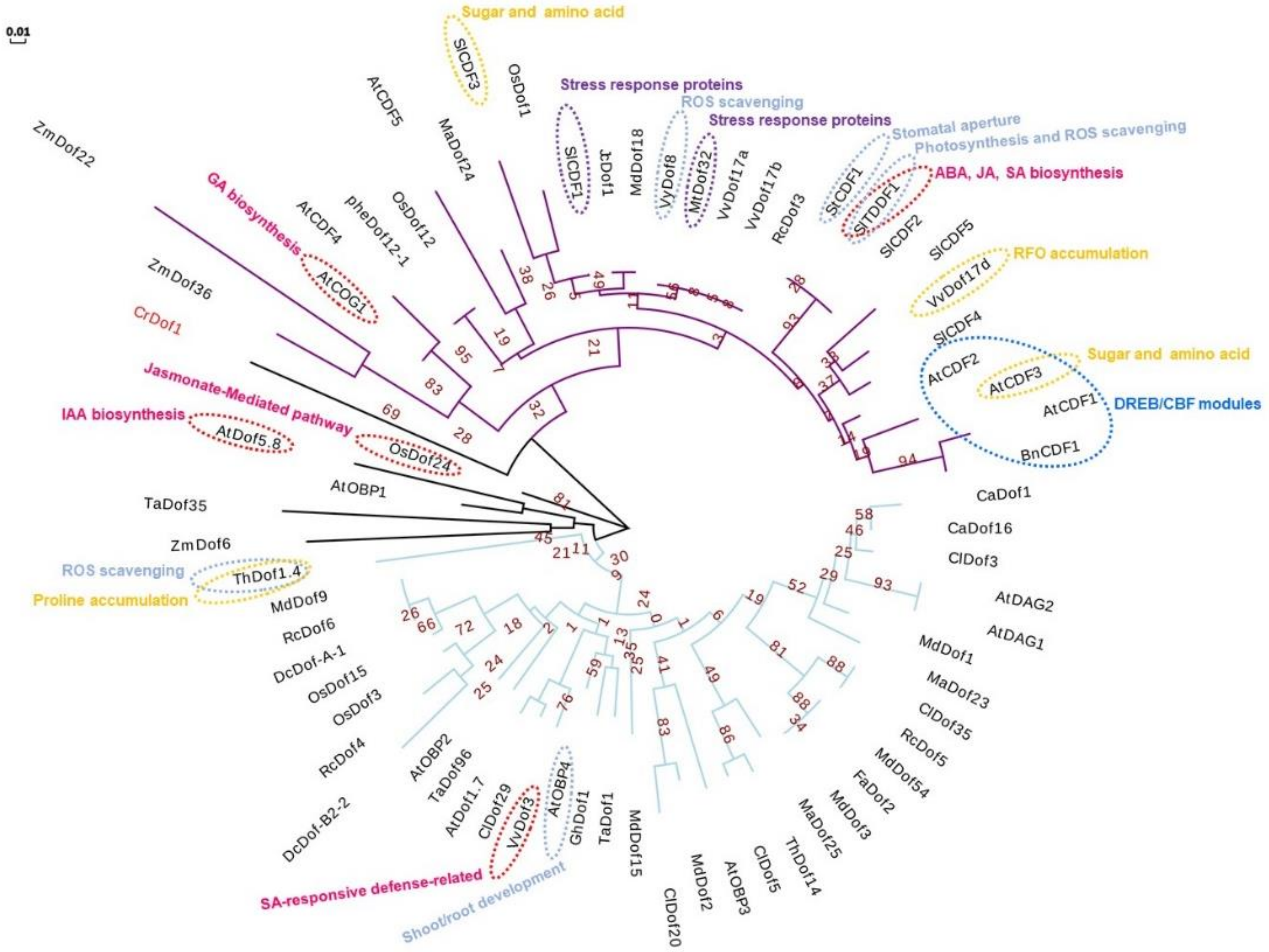

0.01
Sugar and amino acid
SlCDF3
OsDof1
AtCDF5
MaDof24
Stress response proteins
ROS scavenging
Stress response proteins
Stomatal aperture
Photosynthesis and ROS scavenging
ABA, JA, SA biosynthesis
SlCDF1
JrDof1
MdDof18
VyDof8
MtDof32
VvDof17a
VvDof17b
RcDof3
StCDF1
SlTDDF1
SlCDF2
SlCDF5
RFO accumulation
VvDof17d
SlCDF4
ZmDof22
GA biosynthesis
AtCDF4
pheDof12-1
OsDof12
AtCOG1
ZmDof36
CrDof1
Jasmonate-Mediated pathway
IAA biosynthesis
AtDof5.8
OsDof24
AtOBP1
TaDof35
ZmDof6
ROS scavenging
ThDof1.4
Proline accumulation
AtCDF2
AtCDF3
Sugar and amino acid
AtCDF1
DREB/CBF modules
BnCDF1
CaDof1
CaDof16
ClDof3
AtDAG2
AtDAG1
MdDof1
MaDof23
ClDof35
RcDof5
MdDof54
FaDof2
MdDof3
MaDof25
ThDof14
ClDof5
AtOBP3
MdDof2
ClDof20
MdDof15
TaDof1
GhDof1
AtOBP4
VvDof3
ClDof29
AtDof1.7
TaDof96
AtOBP2
MdDof9
RcDof6
DcDof-A-1
OsDof15
OsDof3
RcDof4
DcDof-B2-2
SA-responsive defense-related
Shoot/root development

Figure S1 Neighbour-joining phylogenetic tree of selected stress- and hormone-related plant Dof transcription factors (Tables 1) with *C. reinhardtii* CrDOF1 as outgroup Moreno-Risueno et al. (2007). The physiological function of Dof transcription factor are generally classified into ROS scavenging and others (lightblue), production of compatible solutes (orange), hormone synthesis and signaling (pink), stress response proteins (light purple) and stress-response TFs (blue) and proteins (light purple). The optimal tree with the sum of branch length = 3.47419992 is shown. The tree is drawn to scale, with scale bars representing branch lengths (number of substitutions per site). The analysis involved 69 amino acid sequences. All positions containing gaps and missing data were eliminated. There were a total of 49 positions in the final dataset. Evolutionary analyses were conducted in MEGA7 with Neighbour-joining method and 1000 bootstrap test replicates. Concordantly, other studies encompassing a wider range of plants such as sorghum, rice, and grapes, among others also showed some clusters in common with our analysis (Kushwaha et al. 2011; Noguero et al. 2013). Mention here that most hormone- and stress-related Dofs fall within 3 groups (of 6 total) according to the nomenclature of maybe Noguero et al. (2013).

Dof protein seq use in Fig. S1

>pheDof12-1
MGACRAAAGGGDCLIKLFGKTINPVPDAGDGDVDKDLQHSGSSTTEPKVQENIPQDSTGSPPQPEV
ADTEDSSAVKNSSEDRQQQQGDTANQKEKLKKPDKILPCPRCSSMDTKFCYYNNYNINQPRHFCKN
CQRYWTAGGAMRNVPVGAGRRKSKSASAASHFLQRVRAALPDDPLYTPVKTNGTVLSFSSNTSTLD
LTEQMKHLKEKQIPITQIKNTDDPSIRSCAEGLAKGEDSNQVNLKEKVTADKSANVAQHPCMNGGT
MWPFSCAPPPAYYTSSIAIPFYPAAAAYWGCMVPGAWNTPWPPQSQSQSGSSPSAASPVSTMSNC
FKSRKHPRDGDEEGDAKGNGKVWVPKTVRIDDADEVARSSIWSLIGIKGDKVGTDDGRGCKLAGVFE
PKGEAKTATHTVINSLPFLQGNPAALSRAVTFQEGS
>SlCDF1
MREVKEPEIKLFGKKIVLPENGMILPVIVTGEDSDVGKSMSASEVVTADESSTGSDRDPCLV
DKEGNSSQQDESDDGSEYEKDEADKDRMTRELSEAKLEEKDQNLMMEESENLKSPSENK
TKTHTIDDDSPTVKSSKTEDDQNDASNSQQKTLKKPDKILPCPRCNSMDTKFCYYNNYNVN
QPRHFCKSCQRYWTAGGTMRNVPVGAGRRKNKNSASHCRHIMISEALEAARIDPPNGFSH
PVFKPNGTVLSFGPDLPLCDSMASVLNLAENKTPNGIRNGLYRPENPSGIGGENGDDCSSG
SSVTTSNSMAEGVKNCPPEAVMQTINAFPSPVPCLPGVPWPIPYAAVPFPAISPAGYPMPF
CPPPYWNCNVPGPWSLPWLTPPSPTANQKGSVSAPNSPLGKHSRDGELLKPNNPEGQKN
SEGSVLVPKTLRIDDPDEAAKSSIWSTLGIKYDSVSRGGLFKSLQPKNSEKDHPTTTSPSLQ
ANPAAFSRSLSFQERV*
>SlCDF2
MSEVRDPSIKLFGKTIGMTQQETNCVYLHDDHTTSSPLSIDDEKINLEGEVTQSKQVDELVD
PAADSSIEPETSSGISDDIKMQDADKETLSSKSVEEEDSSEEKALKKPDKLIPCPRCNSMETK
FCYYNNYNVNQPRYFCKNCQRYWTAGGTMRNVPVGSGRRKNKSSSISNYPLQAGRVEAA
AHGMHLPASRTNGTVLTFGSDKPLCDSMVSALNLAENSHNMHRNEYHGSEQRMPTIGNDQ
SNGSCSTASSVTDKESSAGTHDLANWSNFQPFPPQVPYFQGAPWPYSGFPVSFYPAAPY
WGCTVSNPWNVPWLSSNQSVHNNSPTSPTLGKHSRDESKLDPSQSRRRDTTLQDREGER
CVLIPKTLRIHDPNEAAKSSIWSTLGIRNEKIDSTRGTMLFSAFNPKADHRNRELDTSFALQA
NPAALSRSLHFRESTR*
>SlCDF3
MTCDSEIKLFGKILPVVVSGVGRGLSGSDGVIYDGNRNGSDLDRCLEGSKASSVEKDEGSE
YEKQEAEKDNITGELSEAKSEEGDQNQMIEESENPKTPSESESSPKSSTEEDPQAVKSSKT
ENEPTNVTNSEQNNLKKPDKILPCPRCNSLDTKFCYYNNNNVNQPRHFCRSCQRYWTAGG
TMRNLPVGAGRRKNKNLASQYRNISIPEGLLAAGIESPNGLIHHPLFKPNGTILSFGPDLPLC
EPMASALNQAEKRVSTGIQNGSHKSEVKNSSCKGGDSGDECCRGINIPTPNMMVEEGKGE
PHKAVMHSINGIPSPVPCLHGVPWPFPWNAAVPVSAICPIPFPMPFFPTPYWNCSVPPWSN
PWLSPPLRAANEKTSGSDPTSSLGKHSREGDLLKPSNPGGKEQSEQKYSEGSILVPKTLRI
DDPDEAAKSSIWSTLGIKYDSTNRGEFFKALQPKSNDKHNKANTFPVLHTNPAALSRSITFQ
QGA*
>SlCDF4
MTDPAIKLFGRTIQLPDIPDSSGAQGDDSLPGDNNGEEDEEADKDDFGGNLDDDEEEMEILT
GKELQDQNSEPTKTDSIKELPVDNDCSTRPSKSEEEQGEASNSQEKILKKPDKIIPCPRCNS
METKFCYFNNYNVNQPRHFCKSCQRYWTAGGTMRNVPVGAGRRKNKNSIPHYRQISVSET
LSNAQTDYPNGIQQPILAFGSPTPLCESMASVLNIADKTMHNCSQNGFHKPQEPGVPVSYV
VGDNGDDHSRRSSVTSANSEDEVNKTVPDLLKKNCHNFPPYMTYYPGAPWPYPCSPVPW
NSAIPPPGYCPPGFPMPFYPAASYWGYTVAGSWNVPWMSPATVSLIQTPTTSGPISPALGK

HSRDENIQKPLSSMEEPSNESNPEKCLWVPKTLRIDDPGEAAKSSIWATLGIKHDTVDSVGG
SPFSAFQPKNDDNNRVSENSTVLQANPAALSRSVNFNESL*
>SlCDF5
MSEAIAIKDPAIKLFGWTIQLPDFPAPAPEDSSFLAGEVEQELKGLYDDCIDDNEHLTTEDSQ
DQNPIQQRCDIINYYESSTAKTSKSKEEHGETSNSHERNLKKPEKTLPCPRCNSMETKFCYF
NNYNASQPRHFCKNCQRYWTAGGTMRNVPVGAGRRKHKNSVLHYSHVSVSEALSNVRTN
FPTETQHPPLTLNGTILSFDTDKPVSESMVSVLNVADKGMQNCSGNGFQKYKELRIQAGDN
GDDHSDGSSVTAISSKDSDNGLPNTPRKNYNSFPTHLPCFTGAPWPYIWSSVHCRNAVPP
PGYSLPGIPMSFIPATTYWGCTIPGSWNVPWMSPPTASHNQMPLTPDPNSPTSRKHSRDE
NVLKSTGTEEEQRKESDPGKRLWFPKTLRIDDPGEAAKSPIWATLGIKHEVVNSVGGGLLS
DFLPKNDERSCVSENSTLLQVNPAAMSRSLNFNESS
>DcDof-A-1
LPPPTGVLQSSSTTTTTTTNPKPSSENSNPLRCPRCDSSNTKFCYYNNYNLTQPRHFCKTCRRYWTKG
GALRNVPIGGGCRKNNKANISHAISSALEKPTTGKYIRSHILTSENMLQGKASSAYLGGSDHGGLSTHS
LLWTSPQTSHLLALLRSNQAQNPDPNYDCSNIINSVKEEGPFLNGSGGFRLDPYGNNLNNQQQSG
GFIGQNLNNQDQVCDQRASFKSAENYYSDNYAAANLLMGSGHTPSANCSDILESTPVLGGGHQLV
GCWNNASLSRPELPTTDGAYH
>DcDof-B2-2
MMAQDNLNPNPPDDSSGNSQKAACRPAEAPRCPRCDSPNTKFCYYNNYSLSQPRYFCKTCRRYW
TKGGALRNIPIGGGCRKNKKMSSKSCSRFASDSMDSGGSSSYLDHLGGFKFSNIGLSSGMNFQTLDT
NLPRTFSPVQQYQQLSTNSSFGNFSRTLSSPCFSLENQTGSSSGSAFMGLNFPLSSSGIKQVHGSAQL
LQDHDLDIKNNDVVASSIESLSSINQDLHWKLQQQRLGMMMFGEENSITDQKEVLRNTSVIMPPPM
NVVPQKPQPILFENLEISSRVKDQDVHQGVGSSGSGTSGTEWLLDHNAAQPNTAITGMNQAWGNF
YQYTPLP
>JcDof1
MQENNNSNSGSKDSAIKLFGKKIPLKSDSDPPAISCDELPSWERVEEGEAEKTEKDSSAGNVKETSQE
DDTEESSDQETNENPKTPSISEESAKSKISETEKEQNDPANTTTQEKALKKPDKILPCPRCNSMDTKFCY
YNNYNVNQPRHFCKACQRYWTAGGTMRNVPVGAGRRKNKHSASHYRHITISEALQAARIEAPNGT
HNPALKTNGRVLSFGLDTPTCDSMASVLNLADQNVLNGATRNGFHNLEGQKKILGSCKGRENGDD
CSSASSVTVSNSVEEGGKICQQEHIMQNVNGFASPIQCLPGVPWPYPWNSALPPPVFCPPGYPMSF
YPPFGNYGWNIPLLSPQSSSSSTNQKAQSSPNSPTLGKHSRDGDALKQDDLEKEESPKRKNGCVLVP
KTLRIDDPTEAAKSSIWATLGIKNETYSSGGLFKSFQSKNNEKNHVNKTSPVLHANPAALSRSINFHESS
>ThDof14
MSEDGGFDSSSSPTSHDMLTCSRPLIERRLRPPRDQSLKCPRCESTHTKFCYYNNYSLSQPRYFCKTCR
RYWTKGGTLRNIPVGGGCRKNKKPTTTNNGGNGGGGSNSKKSSSSSSSVQSLSSNDHLHDQHNPM
SLLLQNSIGLSSSMMPSGNNNPTELQLSFPHHDQATFSHFSNLIGVHGGLGGDIGFMESKYNAMLEN
NNNNNNNNNNNNGNSCSNIPSRPIDFVENYKLDALVGHDVIRGHDFLGNGAAGTTSVGGGFGNIM
GGINGVDPLSSTGHGHDLSGCGDYSGFCSPFGLASMDHGSNNTNAGGYGAFLDQRLMLPFDGND
HREMPNSIDVKPNATKLLSLQWQDQHQGCSDDRHNNNINGGTSASKGSFGYNNVGGLSSWSGT
GMISGYGPPTTNPLV
>TaDof1
MQDFQSIPGLAGRLFGGAAAADIRRVQGPASRCGVFSQAASAQPEAAVKCPRCESTNTKFCYYNNY
NLSQPRHFCKSCRRYWTKGGVLRNVPVGGGCRKAKRSSSSASAPSTPAATDAKSQRRASASSSSRSN
SGSGSASPTAAAEETTTTETEPPPPPTPSSNSNSNAVSFANRMTNYPFAADVPPLAPIFADQAAALAS

LFAPPPPPPLPVFSFSAEPKMEEAIGSLLLPGQEASQEPEEPTCTSTVADMAPFMSLDAGIFELGDASPA
DYWNGGSCWTDVQDPSVYLP
>VyDof8
MAEAKDPAIKLFGKTIPLPEVATAAAGNDSPSGATVGGGGEDWVDQNRATNSSPEEDCVRAGEEGR
EVDKDTSGGKVTDTRQEDGARSSTSEEFTDPDANSGVNENPKTPSADKETATLKCSKNEEEQSETSIS
QEKTLKKPDKILPCPRCNSMDTKFCYYNNYNVNQPRHFCKNCQRYWTAGGTMRNVPVGAGRRKN
KNSTSHYRHITVSEALQSARTDVPNGIHHPALKTNGTVLTFGSDTPLCESMASVLNLAEKTMRNCTP
NGFHKPEKLRIPVPYGGGENGDDHLSKSSVTASNSKEEAGKTALPDQVMRNCHAFPPQIPCFPGAP
WPYPWNSAQWSPPVPAIPPVPPPAFCPSGFPMPFYPAAAYWGCTVPGAWNIPWVPQPSALNHTA
PSSGPNSPTLGKHSRDENMLKASNFGEEELQKENNSERCLWIPKTLRIDDPGEAARSSIWATLGIKND
KADPISGGRLFKSFQSKADEKNHIAETSPVLQANPAALSRSLNFHESS
>ZmDof36
MLPPHVEMPTTRAAGIKLFGKVITTHYQQQQQPPQPVRHAGAAPASSGRGSGGGPGPDLL
EEVARARAAAAEARLPCPRCLSRDTKFCYFNNYNVNQPRHFCRACHRYWTAGGAIRNVPV
GSGRRKNRPVLLPPPPPPPHAATATTTGSSATISADDSNDRRRSASGSPPPVFTSAFTAP
YYLGSPAQFATSSPAYAAGAAGPPGTAGQCLWLVAATTTTDRSAATETALF
>ZmDof22
MAANLPDADAAGFKLFGKVIQPPDAQKRGADEGDDPTPQTALPPPPPPPPPPPPQQAPAA
GGPGAEPLPCPRCGSRETKFCYFNNYNVRQPRHLCRACRRYWTAGGALRRVASASPGRRR
PRPTARSAPVAAAASSAPAAAEEDSFGGQKDAAGLLCDRERVQFGRQSKRPPLQHARVTE
QCRCRSSKKTCFITASNELVRVLANPTGVLNPSAASLLAPRCAALRAGS
>ZmDof6
MPSSFLTSSSSSASSHLSYLIPARAAPPPPFAMGQGYGATSGGGVANATSAAAAPPPPRQ
GASRNAGAGHPPLPRPPPRQCPRCRSANTKFCYYNNYSREQPRYLCKACRRHWTEGGTLR
DVPVGGGRKNRRGAKGGAAAKGSASAAAAAPAQQGGNVLGADTFPGDLLRQLVQFQPDAA
VGGGGYAIDLSAWHQMVAATAPPPPPPGTGGDVSSLGLGAAGAGAGAEANCVALQYWSED
GMPGLDGAC
>MtDof32
MFMWLKFIFTSDVALQTFLNGSTLSHSFTTCHFSFLSLYNNLLQHLFFSFYYNNRKSFHLFLATKMLETK
DSAIKLFGKTIPILEVRVGDVVVESIDDDHHHFSTNSSNESRNNKDEQDQEIEKNTLGEKPSDEKNEDG
IPIQSTEEFTNPDAASRTDEESITVSTERKAATLKTSKTEEEQDETSNSQDKILKKPDKIVPCPRCNSMDT
KFCYYNNYNVNQPRHFCKNCQRYWTAGGTMRNVPVGAGRRKNKSSSSHYRQITVSEATLQNSRIH
PSVKCNGTILTFGSNSPVCESMASVLKHADKTMQNYTRNGYHKHEELRICVPHTSEEQGEDQSNKSS
VTSTKSTEGATTNVSQEQAMWNDHSFPPQGGYFPHGTPWHLPWNPVQMSSPIPPPAFCPPGFSM
PFYPATTYWGCTMPSAWNIPRQAQPSSPNGANHDSTPNSPTLGKHSREDNMLKSSEGDGKKEISEE
KSLWFPKTLRIDDSEEAE
KSSFWTTLGIKNNNADSVPPRRLFQAFPSKCDEKNHLVQVSSVLQANPAALSRSLHFHETS
>MdDof54
MFTDPTVKQMLQCPPSGQGQFMMMEMKRPWNKSHIEVAPNCPRCASSNTKFCYYNNYSLSQPRY
FCKGCRRYWTKGGSLRNVPVGGGCRKNRRGKATRLSQTDSASLSYFQHHNSSSGTEDDPSGNNQP
GGSNASDIDLAVVFAKFLNHNSTTPDDHYHDPNLVISSSALNDHDGSQNSSKAGDLVEAVDHYLLE
GHQQSQEENVQTFMGINHNDDMNIHEFELQGLPVDEDQVVQDVFWSDDATMSSLA
SSTASFTWQPMVHLQELDCSLPSDDDQMKISTNLCGDNWSTFDFSGFEVFSRS
>GhDof1

MQDIHSIKGGCIFGGGGGGG GGGGGGDKRLRSHHHHNHQALKCPRCDSLNTKFCYYNNYN
LSQPRHFCKSCRRYWTKGGI LRNVPVGGGCRKAKRTKTKASSETTAVAASALPPPEQQHC
DQRKANSHSSSESSSLTATNSNVAVPNQNNNYSSAAGTAE AVTSHSNLIDVSEPKFYGNP
NNLGLEPGLLEQGSDCGIFPEIGSFTSLIT SSNNETMSFG FDKVLNGQALEQGQWQQQHQ
KMMRMGGDEI NGGLLDQTVQVELSNMHSRSDNGFGALDWQGNEDQGLFDLPNDVDKTYWS
QTQWTDQDHH GLYLP
>MdDof1
MKSKNQDFGIMRSIKRNPLSLRTLMIPQKMNKNEFSKQNQTKIPHRITQT
ALCSDILGGIVKTRTFLYFSPHRQSHKGSGEPEKPMEDHDMGSNGCTRSV
LEKRARPQEQLNCPRCNSTNTKFCYYNNYSLTQPRYFCKTCRRYWTEGGT
LRNVPVGGGSRKNKKSTSSSVSSKITIPDLNPPTTLSHFSSSHQNPRVTH
EGQDLNLGFNAIDAHYHGTTMENNNSTNSSSXPNLSAMELLRTGIASRGV
NSYIPTQNMPDHHSNTLYPPSSAGFSLQEYKPTNLGFCVDGLGNRYGGDD
HGTVNGGRILFPFGDHHLKQISSTAXHHEVLDHHQNKGQGNPTGYWNGLL
GGGSW
>MdDof2
MVFSSVPVYLDPPNWQQQQTNHHQQGGGSDQNNPQLPLVPSSEARCTSVD
GGGGLGSIKPGSMCDRARMAKLPQPETALKCPRCESTNTKFCYFNNYNLS
QPRHFCKTCRRYWTRGGALRSVPVGGGCRRNKRSKGGNSSRSKSPGAAGN
SSSSTVNSSNSCSASDNIIGHLTHPPPPHQLPFLPSLHHLPDYGSGDMLG
LNNFGINQPGDVEFQHHQYGSDRFGARPNILGMNNMSSTEHWRSSLLQQQ
FPNFLVNLGQPTSTTHGLYQFDGGDHQNVVQLPISKPLDHSAGNGVTTTQ
MANVKMEQNQALNLSRNFLGSLGNDHQYWGSSTTGTGGNAWTDLSGFTTS
SSTSHLL*
>OsDof1
MDDLAAASPPHPPPPPPESHVPPPPQTPEKDSCEDTGDMRISEEKPCTDQELDADQMNSS
SFNSSSECENQTPSNDEMTGSESKSEAAQTEGGGSSEEKVLKKPDKILPCPRCNSMDTKF
CYYNNYNINQPRHFCKSCQRYWTAGGSMRNLPVGAGRRKSKSSTANYRSILITGSNLAAP
AGDAPLYQLSIKGDQTATAVKFAPDSPLCNSMASVLKIGEQSKNAKPTSTAQPRNGETQT
CPASGTTSDSPRNEPVNGAVSGHQNGIVGHSGVPPMHPIPCFPGPPFVYPWSPAWNGIPA
MAPPVCTAPAEPANSSDNGSTASVQWSMPPVMPVPGYFPVIPSSVWPFISPWPNGAWSSP
WIQPNCSVSASSPTSTSTCSDNGSPVLGKHSRDSKPQGDDKAEKNLWIPKTLRIDDPDEA
AKSSIWTTLGIEPGDRSMFRSFQSKPESREQISGAARVLQANPAALSRSQSFQETT*
>OsDof15
MIQELLGGTTMDQLKGASALNHASLPVVLQPIVSNPSPTSSSSTSSRSSAQATQQRSSSA
TSSPHGQGQGGGAAEQAPLRCPRCNSSNTKFCYYNNYNLTQPRHFCKTCRRYWTKGGALR
NVPIGGGCRKPRPMPAPVAKPPMSCKAAPPLGLGGGPVSWASGQQAATAHLMALLNSARG
VQGHGGSNVHRLLGLDTMGHLQILPGAPNGAGAGTAASLWPQSAPRPVTPPPPHMDSQLG
MGTLGHHDVLSSLGLKLPSSASSSPAASYYSDQLHAVVSNAGRPQAPYDVATASLPCTTA
VTSLPSALSSVSAAAPTSNTVGMDLPPVSLAAPEMQYWNGPAAMSVPWPDLPTPNGAFP*
>OsDof24
MQEQQPETGRRPAQQFATVDLRRPKGYAAAPATPQPGSAATAAAAAGPAATAAAAAAGEG
DPCPRCESRDTKFCYYNNYNTSQPRHFCKCCRRYWTKGGTLRNVPVGGGTRKKSSSSSSS
SSSSSAAAAAPAAKRQKTSKKRRVTTPEPLAATTPVLTEAAADSAAKTTTEATSEKKTTT

STTTTTPPAPDTTSEITTELVVPAVEEDSFTDLLQPDSAAVTLGLDFSDYPSITKSLADP
DLHFEWPPPAFDMASYWPAGAGFADPDPTAVFLNLP*
>ThDof1.4
MGLSSKQVSSDGQDWNQSFLQATSTNTTATTISSIDPIASTMEVPNQQLTPTKRLQNQVQAEPLRC
PRCDSTNTKFCYYNNYNKSQPRHLCKTCKRHWTKGGTLRNVPAGGGRKNKRVRTSNTSSSSLAGITT
PSATKVTPVTSASTATATKSTLSNIMKQHIEGHGFSLPHYSKGMMIKSPATSLLGEGLFNNSNLNKAT
NLNEMLLDATTLSLPIDRSIDLHQFPYTNLSTSSACSKTSMPYSFQSPISLYAYAVDHQPSSTSALEETV
TGGLETSISGSSGSDQHQPWTTMNNTSRFSSSTGAIGMPTSNWGWDDIDKFASVDIAIPWDDDVEI
NP
>TaDof35
MASASASLLPAADSAPDAAEQEQPLAQVSLCSLLTLSQSGQQGGDQKGAASKKNDHGHSQ
QQQPKLECPRCSSTDTKFCYYNNYSTAQPRHYCRACRRYWTHGGTLRKVPVGGACRRRSG
SGKRRRSSAEPHTPSSDSPPPPQPEQQDTLPPLPVFPFLTDGGPIFLPQFDLGLAGFPWT
TPTATDHLYDGLGAPWGGCDGALAPTGAWDDFGGLELTWPLAAGN
>TaDof96
MQEFQSIPGLAGRLFGGAAESAGLLRRGGAAAAQEEVRCPRCDSANTKFCYYNNYNLSQP
RHFCKGCRRYWTKGGLLRNVPVGGGCRKPKRKAAAASSDVEKDASSANSEAKNARSGCSA
GSSSLTSAASSASTNTVIDAGACASAHSSGGSMPFTGLGPSTFMADAPPLQPPAPMFADQ
AAAFASLFGTPLPAFSFSAQRKAEDDVAPAVTLTEQPSSASSTADLAPFSARSTGAVTAS
ASDWPPATVIEAGIFDIAGAVGSDTTSYWNTASWTDPDGTVYLP
>VvDof17a
MLDFKDPAIKLFGKTISLPLNPHLSPTSPPPPPLSSTTSFPDDTSQGLQPPSQDQKPLEGQEFEGKEEDG
TSRQTSEELKDPTASPGVSENPETPSADKETSKDGEQSEISGSQEKTLKKPDKILPCPRCNSMDTKFCYY
NNYNVNQPRHFCKNCQRYWTAGGTMRNVPVGAGRRKNKNSSASQYRHIMVSEALQTARASAAN
GIHHPALGNNGTVLNFGSDGPLCESVASVLNLADKTQNCMQNGFHKSEQRIPASCGGGENGDDHS
SITLTNSAEKGNIAGLEPVVKNFQAFSPHVPCFPGASWSYPWNPAQWSSKIPPPAFCPPGFPISFYPAP
AYWGCTVPGSWNIPCIPPTSSSPIHSALATNHNSPTLGKHARDGEVLNPANPGKEDHQKENNPERG
VWIPKTLRIDDPNEAAKSSIWTTLGIKNDGSNGGSLLKAFQSKGDEKKRIAEMSPVLQANPAALSRSL
NFHERA
>VvDof17b
MAEAKDPAIKLFGKTIPLPEVATAAAGNDSPSGATVGGGGEDWVDQNRATNSSPEEDCVRAGEEGR
EVDKDTSGGKVTDTRQEDGARSSTSEEFTDPDANSGVNENPKTPSADKETATLKCSKNEEEQSETSIS
QEKTLKKPDKILPCPRCNSMDTKFCYYNNYNVNQPRHFCKNCQRYWTAGGTMRNVPVGAGRRKN
KNSTSHYRHITVSEALQSARTDVPNGIHHPALKTNGTVLTFGSDTPLCESMASVLNLAEKTMRNCTP
NGFHKPEKLRIPVPYGGGENGDDHLSKSSVTASNSKEEAGKTALPDQVMRNCHAFPPQIPCFPGAP
WPYPWNSAQWSPPVPAIPPVPPPAFCPSGFPMPFYPAAAYWGCTVPGAWNIPWVPQPSALNHTA
PSSGPNSPTLGKHSRDENMLKASNFGEEELQKENNSERCLWIPKTLRIDDPGEAARSSIWATLGIKND
KADPISGGRLFKSFQSKADEKNHIAETSPVLQANPAALSRSLNFHESS
>VvDof17d
MNGGDLAVSRDPAIKLFGTTIQTQICSNSPQKSLDAPSEITKAEAEDPCAEDSRKPDGSSVSEDGKEE
QQTQVQMSGLQVHKDQGETNSSAQEKVLKKPDKILPCPRCNSLETKFCYFNNYNVNQPRHFCKNC
QRYWTAGGTMRNVPVGAGRRKNKHLASQYRQIMVSSDGVPTTVIEASDSSNQQILSCGETSTTFRP
STASGTVLKFGPEAPLCKSMETVLSIREQKRCAEMRTVNCGGNGEEPSSCASSVSAPSFPENEFPENV
GHKDRPSLPASRSEIHPQLHQPCYPVPPWAFPWNPGWTNVAPVAPPQCSSDTVYAPNNSNPNSV

QWCSRPMLAVPGFCAPTIPLQLVPPSYWGCMPIWGAGTGNISLAGSNDCLSPSSSTSNSCSGNASP
TLGKHSRDAQPAEEQKLEKCVLVPKTLRIIDPDEASKSSIWATLGIKPDQKAPISKGGIFKAFEPKSGAKT
DLSDATQVLEANPAALSRSQTFKEST
>StCDF1
MSEVRDPAIKLFGKTIGMTQQETNCVYLHDDHTTSSPLSIDDDKITLEGEFTQSKQDDEL
VDPTADSSIEPETSSGISDDIKMQDADKETLSSKSIEEEDSSEEKALKKPDKLIPCPRCN
SMETKFCYYNNYNVNQPRYFCKNCQRYWTAGGTMRNVPVGSGRRKNKSSSISNYPLQAGR
VEAAAHGMHLPALRTNGTVLTFGSDKPLCDSMVSALNLAENSHNMNRNEFHGSERRMPAI
GNDQSNGTCSTASSVTDKESSAGTHDLANWNNFQPFPPQVPYFQGAPWPYSGFPVSFYPA
TPYWGCTVANPWNVPWLSSDQSSVQNNSPTSPTLGKHSRDESKIDPSQSRRRDANLQNRE
GERCVLIPKTLRIHDPNEAAKSSIWSTLGIRNEKIDSARGTMLFSAFNPKADHRNREHDT
SFALQANPAALSRSLHFRESTQ*
>CaDof1
MDTANWPQEIVVKPMEEIIGSSKPNNCVERKLVRPQKDQVVNCPRCNSTNTKFCYYNNYS
LSQPRYFCKTCRRYWTEGGSLRNIPVGGGSRKNKKSSSSNNNNNNNNNSNINHVVINNPLM
KKLPDLIVPPLIQHDIEEYPERHFGNRPDNSTVLPQHHHDQNPRKIIHEGSQDLNLGFSS
DFKTITELIQVANYDGGNKDNNNSTNNISTLPPPSEPSSPAFSQLPSLNFSLDHHGLGNN
NVRSGYGFFFPFVGLKQVSNASDHVRDQSTNNGYWNGMLGGGGGGSW*
>CaDof16
MDTSQWPQGIGVVKGVEPSSKAVVLPDQRKPRPQKEQAINCPRCNSTNTKFCYYNNYSLS
QPRYFCKTCRRYWTEGGSLRNVPVGGGSRKNKRSNNNSNNNNNNSSSSNSSSTSSSLSSSK
KLLSDLANPNDLNLTYNPISATAAVATTSTAGNFSNFSEFMALPLIHPANSTSSFMPNNL
YTSSTGLPNLHDLKSSSLNFSLDGFENGYGSLQGGDQEAKLFFPMDDLKINVSTANDQFE
ENREQAAADQSNGFWNGMLGGXXXXXXXXXXXXXXXXXXXXIRGGVYFNFISFVFFKVIF
TG*
>ClDof5
MGLTSLQVCMDSSDWLQGTINEESGMDSSSLSGDMLSCSRPLTERRLRPQHDQALKCPRC
DSTHTKFCYYNNYSLSQPRYFCKTCRRYWTKGGTLRNIPVGGGCRKNKKVSTTKKSNDQQ
QQQQQQQPQIQQPISQNHHHLLHHHHHHHGPSSSSSSSLHIHNPTDLHLSFPDQVQFPHF
NPLIGTTPPNFTIGMLETHHHHHQTRPIDFMDTKMEAIVGNNGHYSTNTDHLAMVGGLNG
DHHITAAATNFHGLCSPYGLSLDGNINQMMIPYDQHQTNEDPNGMDVKPNTKLLALEWQD
QGCSEKVESYGYINGIGSSWNGMMNGYGPSTTNPLV
>ClDof29
MQDPSTFQPIKPQFPEQEQLKCPRCDSTNTKFCYYNNYNLSQPRHFCKNCRRYWTKGGAL
RNIPVGGGTRKNSKRAVAATVKRQPSTSSSSSSTLTNPNPITAPDHNPIGIYGGGLDIPG
SFSSLLASNGQFGNLLEGVDPNQSELKMVELGEFSGSGRKSAAEEQSTAVPENYLGVLQN
GDSNCWTGGGSHGWPDLAIFTPGSSYQ
>ClDof35
MVEISPNCPRCGSSNTKFCYYNNYSLTQPRYFCKGCRRYWTKGGSLRNVPVGGGCRKNRR
PPKPLNLTPSPSNPKFHHSNDGNCHRNHRGLSDHDMSPTIDLAAVYANFVNQKADTPETL
PTANTTSNATGHFSVSGESGGVGCGNYCEFSMQECGLGANDYQVAGCFGLEENNNDHQVD
NIEVAARELQLPPLPGEDMLGGWGNPMIMVNNHHRLQPTRVEMFGMETQDPNLLSGNWSP
IDLSNYDTFSRA
>AtCOG1

MATQDSQGIKLFGKTITFNANITQTIKKEEQQQQQQPELQATTAVRSPSSDLTAEKRPDK
IIPCPRCKSMETKFCYFNNYNVNQPRHFCKGCQRYWTAGGALRNVPVGAGRRKSKPPGRV
GGFAELLGAATGAVDQVELDALLVEEWRAATASHGGFRHDFPVKRLRCYTDGQSC
>AtDof1.7
MQDLTSAAAYYHQSMMMTTAKQNQPELPEQEQLKCPRCDSPNTKFCYYNNYNLSQPRHFC
KNCRRYWTKGGALRNIPVGGGTRKSNKRSGSSPSSNLKNQTVAEKPDHHGSGSEEKEERV
SGQEMNPTRMLYGLPVGDPNGASFSSLLASNMQMGGLVYESGSRWLPGMDLGLGSVRRSD
DTWTDLAMNRMEKN
>AtCDF5
MSKSRDTEIKLFGRTITSLLDVNCYDPSSLSPVHDVSSDPSKEDSSSSSSSCSPTIGPIR
VPVKKSEQESNKFKDPYILSDLNEPPKAVSEISSPRSSKNNCDQQSEITTTTTTSTTSGE
KSTALKKPDKLIPCPRCESANTKFCYYNNYNVNQPRYFCRNCQRYWTAGGSMRNVPVGSG
RRKNKGWPSSNHYLQVTSEDCDNNNSGTILSFGSSESSVTETGKHQSGDTAKISADSVSQ
ENKSYQGFLPPQVMLPNNSSPWPYQWSPTGPNASFYPVPFYWGCTVPIYPTSETSSCLGK
RSRDQTEGRINDTNTTITTTRARLVSESLRMNIEASKSAVWSKLPTKPEKKTQGFSLFNG
FDTKGNSNRSSLVSETSHSLQANPAAMSRAMNFRESMQQ
>AtCDF4
MATQDSQGIKLFGKTIAFNTRTIKNEEETHPPEQEATIAVRSSSSSDLTAEKRPDKIIAC
PRCKSMETKFCYFNNYNVNQPRHFCKGCHRYWTAGGALRNVPVGAGRRKSKPPGRVVVGM
LGDGNGVRQVELINGLLVEEWQHAAAAAHGSFRHDFPMKRLRCYSDGQSC
>AtDAG2
MMNVKPMEQIMIPNNNTHQPNTTSNARPNTILTSNGVSTAGATVSGVSNNNNNNTAVVAER
KARPQEKLNCPRCNSTNTKFCYYNNYSLTQPRYFCKGCRRYWTEGGSLRNVPVGGSSRKN
KRSSSSSSSNILQTIPSSLPDLNPPILFSNQIHNKSKGSSQDLNLLSFPVMQDQHHHHVH
MSQFLQMPKMEGNGNITHQQQPSSSSSVYGSSSSPVSALELLRTGVNVSSRSGINSSFMP
SGSMMDSNTVLYTSSGFPTMVDYKPSNLSFSTDHQGLGHNSNNRSEALHSDHHQQGRVLF
PFGDQMKELSSSITQEVDHDDNQQQKSHGNNNNNNNNSSPNNGYWSGMFSTTGGGSSW
>AtCDF3
MMMETRDPAIKLFGMKIPFPSVFESAVTVEDDEEDDWSGGDDKSPEKVTPELSDKNNNNC
NDNSFNNSKPETLDKEEATSTDQIESSDTPEDNQQTTPDGKTLKKPTKILPCPRCKSMET
KFCYYNNYNINQPRHFCKACQRYWTAGGTMRNVPVGAGRRKNKSSSSHYRHITISEALEA
ARLDPGLQANTRVLSFGLEAQQQHVAAPMTPVMKLQEDQKVSNGARNRFHGLADQRLVAR
VENGDDCSSGSSVTTSNNHSVDESRAQSGSVVEAQMNNNNNNNNMNGYACIPGVPWPYTWN
PAMPPPGFYPPPGYPMPFYPYWTIPMLPPHQSSSPISQKCSNTNSPTLGKHPRDEGSSKK
DNETERKQKAGCVLVPKTLRIDDPNEAAKSSIWTTLGIKNEAMCKAGGMFKGFDHKTKMY
NNDKAENSPVLSANPAALSRSHNFHEQI
>AtOBP1
MPTSDSGEPRRIAMKPNGVTVPISDQQEQLPCPRCDSSNTKFCYYNNYNFSQPRHFCKAC
RRYWTHGGTLRDVPVGGGTRKSAKRSRTCSNSSSSSVSGVVSNSNGVPLQTTPVLFPQSS
ISNGVTHTVTESDGKGSALSLCGSFTSTLLNHNAAATATHGSGSVIGIGGFGIGLGSGFD
DVSFGLGRAMWPFSTVGTATTTNVGSNGGHHAVPMPATWQFEGLESNAGGGFVSGEYFAW
PDLSITTPGNSLK
>AtOBP3
MVFSSLPVNQFDSQNWQQQGNQHQLECVTTDQNPNNYLRQLSSPPTSQVAGSSQARVNSM

VERARIAKVPLPEAALNCPRCDSTNTKFCYFNNYSLTQPRHFCKTCRRYWTRGGSLRNVP
VGGGFRRNKRSKSRSKSTVVVSTDNTTSTSSLTSRPSYSNPSKFHSYGQIPEFNSNLPIL
PPLQSLGDYNSSNTGLDFGGTQISNMISGMSSSGGILDAWRIPPSQQAQQFPFLINTTGL
VQSSNALYPLLEGGVSATQTRNVKAEENDQDRGRDGDGVNNLSRNFLGNININSGRNEEY
TSWGGNSSWTGFTSNNSTGHLSF
>AtDAG1
MDATKWTQGFQEMINVKPMEQMISSTNNNTPQQQPTFIATNTRPNATASNGGSGGNTNNT
ATMETRKARPQEKVNCPRCNSTNTKFCYYNNYSLTQPRYFCKGCRRYWTEGGSLRNVPVG
GSSRKNKRSSTPLASPSNPKLPDLNPPILFSSQIPNKSNKDLNLLSFPVMQDHHHHALEL
LRSNGVSSRGMNTFLPGQMMDSNSVLYSSLGFPTMPDYKQSNNNLSFSIDHHQGIGHNTI
NSNQRAQDNNDDMNGASRVLFPFSDMKELSSTTQEKSHGNNTYWNGMFSNTGGSSW
>AtCDF2
MADPAIKLFGKTIPLPELGVVDSSSSYTGFLTETQIPVRLSDSCTGDDDDEEMGDSGLGR
EEGDDVGDGGGESETDKKEEKDSECQEESLRNESNDVTTTTSGITEKTETTKAAKTNEES
GGTACSQEGKLKKPDKILPCPRCNSMETKFCYYNNYNVNQPRHFCKKCQRYWTAGGTMRN
VPVGAGRRKNKSPASHYNRHVSITSAEAMQKVARTDLQHPNGANLLTFGSDSVLCESMAS
GLNLVEKSLLKTQTVLQEPNEGLKITVPLNQTNEEAGTVSPLPKVPCFPGPPPTWPYAWN
GVSWTILPFYPPPAYWSCPGVSPGAWNSFTWMPQPNSPSGSNPNSPTLGKHSRDENAAEP
GTAFDETESLGREKSKPERCLWVPKTLRIDDPEEAAKSSIWETLGIKKDENADTFGAFRS
STKEKSSLSEGRLPGRRPELQANPAALSRSANFHESS
>AtCDF1
MLETKDPAIKLFGMKIPFPTVLEVADEEEEKNQNKTLTDQSEKDKTLKKPTKILPCPRCN
SMETKFCYYNNYNVNQPRHFCKACQRYWTSGGTMRSVPIGAGRRKNKNNSPTSHYHHVTI
SETNGPVLSFSLGDDQKVSSNRFGNQKLVARIENNDERSNNNTSNGLNCFPGVSWPYTWN
PAFYPVYPYWSMPVLSSPVSSSPTSTLGKHSRDEDETVKQKQRNGSVLVPKTLRIDDPNE
AAKSSIWTTLGIKNEVMFNGFGSKKEVKLSNKEETETSLVLCANPAALSRSINFHEQM
>AtDof5.8
MPSEFSESRRVPKIPHGQGGSVAIPTDQQEQLSCPRCESTNTKFCYYNNYNFSQPRHFCK
SCRRYWTHGGTLRDIPVGGVSRKSSKRSRTYSSAATTSVVGSRNFPLQATPVLFPQSSSN
GGITTAKGSASSFYGGFSSLINYNAAVSRNGPGGGFNGPDAFGLGLGHGSYYEDVRYGQG
ITVWPFSSGATDAATTTSHIAQIPATWQFEGQESKVGFVSGDYVA
>AtOBP2
MGGSMAERARQANIPPLAGPLKCPRCDSSNTKFCYYNNYNLTQPRHFCKGCRRYWTQGGA
LRNVPVGGGCRRNNKKGKNGNLKSSSSSSKQSSSVNAQSPSSGQLRTNHQFPFSPTLYNL
TQLGGIGLNLAATNGNNQAHQIGSSLMMSDLGFLHGRNTSTPMTGNIHENNNNNNNENNL
MASVGSLSPFALFDPTTGLYAFQNDGNIGNNVGISGSSTSMVDSRVYQTPPVKMEEQPNL
ANLSRPVSGLTSPGNQTNQYFWPGSDFSGPSNDLL
>AtOBP4
MQDIHDFSMNGVGGGGGGGGRFFGGGIGGGGGGDRRMRAHQNNILNHHQSLKCPRCNSLN
TKFCYYNNYNLSQPRHFCKNCRRYWTKGGVLRNVPVGGGCRKAKRSKTKQVPSSSSADKP
TTTQDDHHVEEKSSTGSHSSSESSSLTASNSTTVAAVSVTAAAEVASSVIPGFDMPNMKI
YGNGIEWSTLLGQGSSAGGVFSEIGGFPAVSAIETTPFGFGGKFVNQDDHLKLEGETVQQ
QQFGDRTAQVEFQGRSSDPNMGFEPLDWGSGGGDQTLFDLTSTVDHAYWSQSQWTSSDQD
QSGLYLP

>FaDof2
MFTAPVLEQMLQCQSGQPLIMNSQKPSWKPHVEIAPNCPRCASSNTKFCYYNNYSLSQPR
YFCKGCRRYWTKGGSLRNVPVGGGCRKNRRSRSARLSQADRISMSYYRRDSSSSSGDDLS
EQHSKEGANGSDIDMAAVFANFLNQNSSNGHDPNLVGSESIPDDVHDANALSDHSQNSSN
PAEDLLESVELLQGLVLPDHDLQQSHEEDNIHNVMGNHQSHQDLSIHEFGLQGLLDDEDV
FWSDTTASLTSNTTSFNWQELDNSFPSDDDDHMKISTTTTNLCSENWSSFDFSGLGVFSG
>OsDof3
MAPAAGDDAVVPRKGAGGGGTTTPPPPPPAQQQQQQPLPPPPPQEQGLRCPRCDSPNTKF
CYYNNYSLSQPRHFCKTCRRYWTKGGALRNVPVGGGCRKNKRSRSAAAASRLSLNLPTVE
GIGGAAADPAAAAAARLGFLGGGTTMMMSSSTSPLGGAAAAVADFQQGGAVGMLPLPRLQ
SPAGGGVGHHQYVPFGEWPSGDIAGGNAVNGGGGHGAVSSTIASSIESLSFINQDLHWKL
QQQRLATMFLGPPGSASAAAAAAQANDGGGNGAQATASGHAAGGGGGAFMHMAGSVPSME
AAMPSATSWFMDSCSYGLPSPPPPATAAVAATTSSNLNSGGRSSGGDDNATSNCGSAIPA
WGDISTFAMLP*
>OsDof12
MGECKVGGGGGGGDCLIKLFGKTIPVPEPGACAAGDVDKDLQHSGSSTTEPKTQENTVQD
STSPPPQPEVVDTEDSSADKNSSENQQQQGDTANQKEKLKKPDKILPCPRCSSMDTKFCY
YNNYNINQPRHFCKNCQRYWTAGGAMRNVPVGAGRRKSKSVSAASHFLQRVRAALPGDPP
LYAPVKTNGTVLSFGSDLSTLDLTEQMKHLKDKFIPTTGIKNTDEMPVGLCAEGLSKTEE
SNQTNLKEKVSADRSPNVAQHPCMNGGAMWPFGVAPPPAYYTSSIAIPFYPAAAAAVAAY
WGCMVPGAWNAPWPPQSQSQSVSSSSAASPVSTMTNCFRLGKHPRDGDEELDSKGNGKVW
VPKTVRIDDVDEVARSSIWSLIGIKGDKVGADHGRGCKLAKVFESKDEAKASTHTAISSL
PFMQGNPAALTRSVTFQEGS*
>MaDof23
MCYPWIAAPKFFLWGCGCCQDIGLVKSMEMVASSSSTVTTTTATASSTFSAARPQVTERR
IRPQKEQALHCPRCNSTNTKFCYYNNYSLTQPRYFCKTCRRYWTDGGSLRNVPVGGGSRK
NKRPSLSSAAATTSATSATTSSTAATSSIKFHDGQDLNLAFPHHGLPDNPCNAVGALSAM
ELLRSGMTMRGLSPFVTMPLPEYPTGFELQEFRAPALNFPMEAIAGGEGREGSSGGYGAL
QGVQESAGGRLLFPFEDVKPVVHTSTAAEFENSRGHGGHPPGFWNGMIGGGGSW
>MaDof24
MSELGDAAIKLFGKIITLQNSTPEERPSDAGGWKEKAARKPEKILPCPRCRSSDTKFCYY
NNYNVNQPRHFCRNCQRYWTAGGAMRNVPVGAGRRKNRNTSHDRHVVLRFGSEAAGNIGD
EQSSKESEGTASVPVPFYPAVAEWSCTVPPGAWSTPWLSPVESSSPESSSPTIRKRSRMD
DPEEAARSSVLKAFQRKVDVKDHGMEAPLLLHVNPAALSRSLSFQESSKRSAAVGERRSG
LY
>MaDof25
MQIFMESSDWLKSVVREDGGMDFSSTPGELLSCSRLPVGDRRLRPQQEQAKCPRCDSTHT
KFCYYNNYSLSQPRYFCKACRRYWTKGGALRNVPVGGGCRKNKRPAAKKAAEPHRQPAFS
LPGATDLQSSFNGAQPQQFPSLTEIPGNLNFAECKYDSELGSFSSGLHPMRTKYGAMQGS
SQSFVFVSEGDIGLGVATQGFPSDDNTREVFMDGSHAVLMQACQRIEDPFGQNRNTNTEF
LRLLGRNSGVQMVDVTQWGTPTASDHGPES
>RcDof3
MSEPKDPAIKLFGKMIPFPEISTSTAAAASATTCSADDDCQDRERPSCASSSLEEGDTTG
DGEERESDKDAEGETLAESKQENGSEESSNPDSISGITENPKTSSVEKDDTSKTSKTEEE

QSDTSNSQEKTPKKPDKILPCPRCNSMDTKFCYYNNYNVNQPRHFCKNCQRYWTAGGTMR
NVPVGAGRRKNKNSASHYRHITVSEALQNVQNDIPNGIHHPALKSNGTFLTFGSDTPLHE
SVASALNLADKTVQNCTRNGFHKPEALRIPVSYAGVEHGDAHSKGSLATASNSKDEASKS
GPQATVIQNCQGFPPQIPCYPGTPWPYPWNSAQWSSPVPPTAFCPPGFPMPFYPTTAYWG
CTVPGTWNMSWIPQPPSPNETASTSGPNSPTLGKHSRDDNMLKPSNSGETPEKEKSAERC
LWIPKTLRIDDPGEAAKSSIWTTLGIKNDRPDSIGGRGLFKAFESKGNEKNHAAETSSVL
QANPAALSRSLKFRESS
>RcDof4
MMSPNNLQGKQATNKEDNQSSGNRKSTASARPQEQALKCPRCDSPNTKFCYYNNYSLTQP
RHFCKTCRRYWTKGGALRNVPIGGGCRKNKKMKSSSRLSGDSKDSSGSSEIGGFKFFHGI
SPAMDFNLGGLSFPRLSPSSGGLYNQFSSFGDMSATSAAAAVSVTNPCFNLDPSGSSPVG
SLMGFSYPISSVTSGFSGAIQDVGTGGGGSGSMNVHTNLASSIESLSTINQDLHWKLQQQ
RLAMLFGGDDHKDNSVSSLAVENQAQKPQPILFQNLEISKPEVSGVGNSRKEADTATEWF
FGNSYRQVNPTPTNSNSNGNDNTAGASNWNGVQAWGDLHQYNGLP
>RcDof5
MDRGWKPNVEISPNCPRCGSSNTKFCYYNNYSLTQPRYFCKGCRRYWTKGGSLRNVPVGG
GCRKNRRGKSLRLSSTDVAQSKSLGYGGVTADSTRHVHGYKGINPSIESCSSSTRSNGSH
IDLALVYANFLNPQQPDPKTASSGFEMQELGSDHFDPSLDFSGISNVNLDSTTVQLHDIG
DHNFAGCIPAISDSSMGDPPSDNHQLMHYCGVDSSSKHKQEANDHKIPQCTSITHDPINY
GLPPLPGEEIGSQEILWPSSHMMEHSHTLQVTQQQAVLGPDQTQDPNTLLFGNWSPFDLS
SDDAFSRS
>RcDof6
MIQELLGGAGLIGGETKISIAGTILEGTPSHSPSLSPSSSTTTTSATAATATATTTTANS
TTSSTSENLRCPRCDSSNTKFCYYNNYNLTQPRHFCKTCRRYWTKGGALRNVPIGGGCRK
NKNTVVSASVAKSSNNKMKTMVSEIGRSSFGNVFDHELPSSSPIMWASPHNSHLLTLLRA
NHNPNPNSTTLSNSLGVKEETGCMIGTQMMAETTGATGALNARTLGMDHLSQVPSLGLCS
PFWKNSQNQAQHHQQNGFIVGEAQNSGIQELYQRLRSSTNTYYTDNSSVVLSNVGSSSTS
SSNILESSPVAGGELGYWNPTFSWSDLPTTNGAYP
>MdDof3
MFIAPSVKQILQCPPSGQEQFMMMEMKTPWNKSHIEIAPNCPRCASSNTK
FCYYNNYSLSQPRYFCKGCRRYWTKGGSLRNVPVGGGCRKNRRGKAARLS
QADRGSLSYFQHHNSSTTTEDDLAGNTQPGGSNVPDIDLAVVFAKFLNHN
STTPDGHDHDPNLVISSSALNDRDGSHNSSKAADDLVEAVGHHYLLEGHQ
SQEENVQTFMGINHNDDMNIHEFELQGLLGDDEQVVQDVFWSDDATTSLT
SSTASFTWQPMMDLQELDCSLPSDDDQMKISTNLCGDNWSSFEFSGFEVF
SRS

>VvDof3
MQDPSTFQPMKTHFPEQEHLKCPRCESTNTKFCYYNNYNLSQPRHFCKNCRRYWTKGGAL
RNIPVGGGSRKNTKRSSNPKRPSSSSSTSSVSSPPVSSTSNAAAQKSEPSGIYVTAADQD
RRMLDISGSFSSLLASNGQFGSLLESLNPTGSVRGVPLSAFSETPNSDSGRTPAMELQSN
GNSESFLGIQTGDSNCWNGGNGWPDLAIYTPGSTFQ
>MdDof9
MQSFHSFAPQEYFANKFPYNLSLKTQDPQRRVNQKTKMIQELFGGAGLNN

IAGGGERKSLSLHGGTTPSPSLSSSPSPSCSTTTTATTTTTTGLSNTENL
RCPRCDSSNTKFCYYNNYNLTQPRHFCKTCRRYWTKGGALRNVPIGGGCR
KNKSVTVSTASIGKTAAGKLKTVVSEIGRSGLGGGFEQEVQASPIQWGSP
QNSHILALLRSSHQNPNPNQLCNSVNVKEEGGMNNMIGSRMMTTEPAAMS
TARTLGLDPVCEAPSLGLCSSFWRNNNNQNQPPPSHHQQNGFIVGHEVQS
SNIGIQELFQRLRSTTSQSGSYYSDHLNNVVMSSSSSSLSSSSTSSILDS
APVVGGGEMGYWNPAFTAWSSDLPTTNGAYH
>MdDof15
MLSNCDHEKMALLISSTTNEWPQLQQDIQIDDIHHQKGLNNNNNNVRSSS
TSSPANRVMEKPGQEQLQLQQQALKCPRCDSSNTKFCYYNNYSLSQPRHF
CKACKRYWTRGGTLRNVPVGGGCRKNKRVKRPGSTSSTSSAMDGGATSSS
SSVPSTTAAANNNSNPNSQPSHIDQINPLFYGLSSNPFMDTASGFDLHQL
NHHAQLGLGFSSSSGTNMMSSDNYRNRFNPASSSSLPSNYSSMFSASPST
TVPTMASVLASKFINGGAFKDIQARGPHQQNQYFQSLLPFEDLQIAAGNN
GEVGGAAKDVKVEDHNGQNRLQWNNNNGSSNVNGNMNCQNQISEQMGLSD
PTPYWNSSTLGSNWHDPANLGSSVSSLI
>MdDof18
MLETKDPAIKLFGKKIALPADIEDLGFARPVEKEVEVESEEEDEEEEDEE
TEQDSPSRKSTESIEQDDGSPPKTEDPANLEILPDSNVNPKTPSTDEASA
LSKTAQTEKEPSTENSEEKALKKPDKILPCPRCNSMDTKFCYYNNYNVNQ
PRHFCKACQRYWTAGGTMRNVPVGAGRRKNKNSASHYRHFTITEALQAAR
IDAPNGAHHPALKSNGRVLSFGVDVPICDPMASVLNLADKNVSSSTRNGL
HKGEEQVFRAPCKSRENGDDSSSASSVNVSKPHLVNVNGFSSQIPCLPGV
PWPYPWNAAVPPPAFCPPGFPLSFCAAPYWNCGVPGTWNVPWFGPQPATP
NQKSPHSSSNSPTLGKHSRDEDVVRPENLEKEEPSRQKNGCVLVPKTLRI
DDPSEAAKSSIWATLGIKNDSISGKGLFKAFQPKSDQKKTVPETSPAMRA
NPAALSRSLNFHESS
>BnCDF1
MLESKDPAIKLFGMKIPLSSTVFEAEEYELSAEQNQNETLTDQSDKEKNLKKPTKILPCPRCNSMETKFC
YYNNYNVNQPRHFCKACQRYWTSGGTMRSVPVGAGRRKNKNNSSSHYRHVTINETNGPVLSFNLR
DDHKTLEKNNHQPVDNSRTRNNEMNGLRCIPGVSWPYTWNHGFYPVYPYWNVPMVSSSPYSTPN
STLGKHSREEDETIKRKQMNGSVLVPKTLRIDDPNEAAKSSIWTTLGIKNEVMFKGFDSKKEVSINKEET
ETSLVLCANPAALSRSVNFHEQM
>ClDof3
MGIDYCGFDDQFAGGGVVVKQMEGGGSSSSSKPNNNNNNNNNNNGNLLERKARPQKEQALN
CPRCNSTNTKFCYYNNYSLSQPRYFCKACRRYWTEGGSLRNVPVGGGSRKNKRSSLSSSA
DKKINASDQNPKIIHHAHQDLNLAIFPPNNNNNNNNTPPIPSSTSTSSSSHLLGSFMTAAP
AAGVFNGGGGFGLSELKPSSLSFSLEGFENGGGYGDLHHNHHHHHHHHHHHQDQTAVMFP
MEELKQSNGRGEFEENRGGGGHGAGDNNSGGGFWNGMLGGGSW
>ClDof20
MVFPSIPSNYLDPTTTHQWHQQTASQQSGSSSFQLAGSPAPPQVRPVSMADRARMANLPV
PETALKCPRCESTNTKFCYFNNYSLSQPRHFCKTCRRYWTRGGALRSVPVGGGYRRNTKR
TKSTSKSPVNSQCQPTTTEVSGGEAMRLNYNNAISTQNEGAVDSTHHPIGIANFLGFDQI
QWRPQLQLHQTQQSPLLVNSARLLGNGGIEASPSYSIVRQIQKKMEATTKMEEDGQQVEM

NVAKPFWGVVGGSDHRHYWSGFNPSSSSHS
>SlTDDF1
MVVDSVRNSFDLLCGARTKPVXFLSIDQINLEGEVTQSKQVDELVDPAADSSIEPETSSGISDDIKMQD
ADKETLSSKSVEEEDSSEEKALKKPDKLIPCPRCNSMETKFCYYNNYNVNQPRYFCKNCQRYWTAGG
TMRNVPVGSGRRKNKSSSISNYPLQAGRVEAAAHGMHLPASRTNGTVLTFGSDKPLCDSMVSALNL
AENSHNMHRNEYHGSEQRMPTIGNDQSNGSCSTASSVTDKESSAGTHDLANWSNFQPFPPQVPY
FQGAPWPYSGFPVSFYPAAPYWGCTVSNPWNVPWLSSNQSVHNNSPTSPTLGKHSRDESKLDPSQ
SRRRDTTLQDREGERCVLIPKTLRIHDPNEAAKSSIWSTLGIRNEKIDSTRGTMLFSAFNPKADHRNREL
DTSFALQANPAALSDHFIFVRVHDXYKTYNMIHGCLVPIIHVKSLAKCR
IKLHTIDLCGXGPSSDPAHSRFLCLGGEGDSMHSVYKVL*
>CrDof1
MVDGGSRAASGQLDDWAAGVAADLDQGEGDRAGARRRPARDASPAPDARKVTTFTNKKRPASD
RDSSPEEDDEEQAQKGSLKADGTRPKLPRPDKKEACPRCNSMDTKFCYYNNYNIKQPRFYCKTCQRY
WTAGGTLRNIAPGSGR
